# The Structure of Oxygen Vacancies in the Near-Surface of Reduced CeO_2_ (111) Under Strain

**DOI:** 10.3389/fchem.2019.00436

**Published:** 2019-06-18

**Authors:** Zhong-Kang Han, Lei Zhang, Meilin Liu, Maria Verónica Ganduglia-Pirovano, Yi Gao

**Affiliations:** ^1^Shanghai Institute of Applied Physics, Chinese Academy of Sciences, Shanghai, China; ^2^Center for Innovative Fuel Cell and Battery Technologies, School of Materials Science and Engineering, Georgia Institute of Technology, Atlanta, GA, United States; ^3^Institute of Catalysis and Petrochemistry, Spanish National Research Council (CSIC), Madrid, Spain; ^4^Zhangjiang Laboratory, Shanghai Advanced Research Institute, Chinese Academy of Sciences, Shanghai, China

**Keywords:** CeO_2_, density functional theory, oxygen vacancy, strain, surface structures

## Abstract

Strain has been widely recognized as important for tuning the behavior of defects in metal oxides since properties such as defect configuration, electronic structure, excess charge localization, and local atomic distortions may be affected by surface strain. In CeO_2_, the most widely used promoter in three-way catalysts and solid state electrolyte in fuel cells, the behaviors of oxygen vacancies, and associated Ce^3+^ polarons are crucial in applications. Recent STM and AFM investigations as well as DFT-based calculations have indicated that in the near-surface of CeO_2_ (111), at low temperatures and vacancy concentrations, subsurface oxygen vacancies are more stable than surface ones, and the Ce^3+^ ions are next-nearest neighbors to both types of vacancies, which can be explained by the better ability of the system to relax the lattice strain induced by vacancy formation as well as by the excess charge localization. The results also revealed that the interaction between first-neighbor vacancies is repulsive. In this work, the relative stability of surface and subsurface oxygen vacancies at the CeO_2_ (111) surface under in-plane strain is investigated by means of DFT+*U* calculations. The tensile strain favors isolated surface vacancies with next nearest neighbor polarons, whereas isolated subsurface vacancies with nearest neighbor polarons are energetically favored under compressive strain. In addition, the formation of both surface and subsurface dimers is favored over having corresponding isolated species under compressive strain, which implies the possibility of controlling the formation of vacancy clusters using strain. In many applications, ceria is employed as a supported thin film or within a heterostructure in which ceria can be strained, and this study shows that strain can be a useful handle to tune properties of such materials.

## Introduction

Ceria is widely used as solid oxide fuel cell electrolyte (Inaba and Tagawa, 1996) and in catalytic applications (Trovarelli, 1996; Fu et al., 2003; Vayssilov et al., 2011; Montini et al., 2016) largely due to its facile oxygen vacancy formation and diffusion, either within the bulk or at its surfaces. It has been previously reported (Skorodumova et al., 2002), and later supported by subsequent research (Ganduglia-Pirovano et al., 2007, 2009; Li et al., 2009; Shoko et al., 2010; Jerratsch et al., 2011; Paier et al., 2013), that the formation of an intrinsic neutral oxygen vacancy in ceria is accompanied by the formation of two nearby polarons (Ce^3+^ or ${\text{C}{\text{e}}^{\text{′}}}_{\text{Ce}}$ in Kröger-Vink notation), which result from the transfer of two electrons originally residing in 2*p* states of the missing oxygen ion to Ce 4*f* states of two cations. For oxygen vacancies in the near-surface of CeO_2_ (111), it has been predicted from Density Functional Theory (DFT) calculations that the two Ce^3+^ polarons are not necessarily NN (Nearest-Neighbor) to the oxygen vacancies, but rather prefer to locate at NNN (Next-Nearest-Neighbor) cationic sites (Ganduglia-Pirovano et al., 2007, 2009; Li et al., 2009; Paier et al., 2013), which has been later validated in Scanning Tunneling Microscope (STM) experiments (Jerratsch et al., 2011). The preference for the NNN positions upon localization of the excess charge has been explained by the better ability of the system to relax the lattice strain induced by the presence of the vacancies as well as by the excess charge localization; a Ce^3+^ ion is more spacious than its Ce^4+^ counterpart and at NN sites Ce^3+^ – O bonds would be compressed (Ganduglia-Pirovano et al., 2009). Moreover, the preferred sites for oxygen vacancies in the near-surface of CeO_2_ (111) (Esch et al., 2005; Castleton et al., 2007; Torbrügge et al., 2007; Ganduglia-Pirovano et al., 2009; Li et al., 2009; Jerratsch et al., 2011; Murgida and Ganduglia-Pirovano, 2013; Sutton et al., 2015) as well as the type of interactions between them (Namai et al., 2003; Esch et al., 2005; Torbrügge et al., 2007; Conesa, 2009; Murgida and Ganduglia-Pirovano, 2013; Kullgren et al., 2014, 2017; Han et al., 2018), have also been assessed. Under zero applied stress, isolated subsurface vacancies are more stable than surface ones, and the interaction between nearest neighbor vacancies at the surface or in the subsurface is repulsive.

As for isolated oxygen vacancies in bulk ceria, most theoretical studies reported that the NNN Ce^3+^ locations are also preferred under zero applied stress (Kullgren et al., 2012; Allen and Watson, 2014; Murgida et al., 2014; Wang et al., 2014; Grieshammer et al., 2016; Han et al., 2017), i.e., at the calculated equilibrium volume of CeO_2_. Moreover, it has been demonstrated that the ground-state configuration varies whether tensile or compressive stress is applied (Arapan et al., 2015), namely, at large volumes, both Ce^3+^ ions are in the second coordination sphere of the vacancy, and at small volumes, they are in the first. Additionally, it has been experimentally found that the lattice parameter of reduced ceria in the fluorite-type cubic structure is expanded (Rossignol et al., 2003), which has been consistently reproduced in DFT calculations (Arapan et al., 2015) that predict that the averaged lattice parameter over different configurations for the pair of Ce^3+^'s is slightly larger than the equilibrium lattice parameter of perfect ceria.

Furthermore, for doped ceria with three-valent dopants (Andersson et al., 2006; Wang and Cormack, 2012; Grieshammer et al., 2016), the dependence of the location of the dopants on the dopant's ionic radius has been reported, in which larger ions such as La prefer NNN sites to vacancies, and smaller ions such as Gd prefer NN sites. It has been argued that it is the balance between repulsive elastic and attractive electronic contributions to the interaction between dopants and vacancies what determines the NN or NNN site preference (Andersson et al., 2006), and, as in the case of undoped ceria mentioned above, applied tensile or compressive stress can change the site preferences (Wang and Cormack, 2012). In addition, changes in the lattice parameter upon ceria doping have been reported, as, for example, the one of Gd-doped ceria (Artini et al., 2014; Žguns et al., 2019) that shows a maximum as a function of Gd content.

Strain in ceria has received considerable attention because oxygen vacancy migration can be modified through strain, which is most relevant for tuning ion conduction in ceria-based applications (De Souza et al., 2012; Hinterberg et al., 2013; Sun et al., 2015). It is fair to say that it is practically inevitable to avoid that ceria surfaces will experience strain effects, either induced by the lattice mismatch when created as a thin film on a substrate (Duchon et al., 2013; Luches et al., 2014), or when reduced (Grieshammer et al., 2016), particularly due to the higher stability of oxygen vacancies in the near-surface as compared to deeper layers (Duchon et al., 2013; Murgida and Ganduglia-Pirovano, 2013; Sutton et al., 2015; Olbrich et al., 2017) that are accompanied by the formation of Ce^3+^ ions which induces a near-surface lattice expansion. For reduced ceria nanoparticles, mainly expansions of the lattice have been observed, which were associated to the presence of Ce^3+^ cations (Rossignol et al., 2003; Wang and Cormack, 2012; Allen and Watson, 2014; Arapan et al., 2015), but also lattice contractions have been reported (Wang and Cormack, 2012), which were attributed to the additional pressure caused by the surface tension between the crystallite and the ambient atmosphere as the nanoparticle size decreases. Despite these efforts, the effect of applying a tensile or compressive stress to the reduced CeO_2_ (111) surface has not yet been comprehensively studied.

In this article, using DFT-based methods, we systematically investigate the effect of applied stress on the reduced CeO_2_ (111) surface and address how strain affects the relative stability of isolated surface and subsurface oxygen vacancies, the formation of vacancy pairs, and the localization of the excess charge. It turns out that, depending on strain, surface vacancies can be more stable than subsurface ones, the interaction between first nearest neighbor vacancies in the near-surface can be attractive, and different localized charge distributions can be attained.

## Methodology

First-principles spin-polarized DFT-based calculations were carried out employing the VASP (Vienna *Ab-initio* Simulation Package) code. The DFT+*U* methodology (Dudarev et al., 1998) was used with the generalized gradient approximation (GGA) of Perdew-Burke-Ernzerhof (PBE) (Perdew et al., 1996), and an effective *U-*value of 5.0 eV. The value of the *U* parameter—necessary to describe localized electrons associated to Ce^3+^ ions—lies within the range of suitable values to describe reduced ceria-based systems (Castleton et al., 2007). We used projector augmented wave (PAW) potentials (Kresse and Joubert, 1999) with Ce (4*f*, 5*s*, 5*p*, 5*d*, 6*s*) and O (2*s*, 2*p*) electrons as valence states, and a plane-wave cutoff of 400 eV. The CeO_2_ (111) surface was modeled using three O-Ce-O trilayers separated by 15 Å vacuum space (i.e., a nine atomic layer slab) with both 5 × 5 and 2 × 2 periodicities. A 1 × 1 × 1 (3 × 3 × 1) Gamma-centered Monkhorst-Pack grid was used for the *k*-point sampling of the Brillouin zone of the 5 × 5 (2 × 2) slab. Stress ranging from −5% (compressive) to +5% (tensile) has been applied to the CeO_2_ (111) surface, i.e., to the two vectors which define the surface unit cell. It should be mentioned that for compressive stress, experiments show that CeO_2_ transforms from a cubic fluorite-type structure to an orthorhombic cotunnite-type (PbCl_2_) structure at a pressure of 31 GPa (Duclos et al., 1988). However, in this study, the underlying fluorite lattice is prevented from undergoing a phase transition. In all geometry optimizations, all atoms in the bottom trilayer were kept fixed after stress had been applied, whereas the rest of the atoms were allowed to fully relax. For all strained surfaces, the interlayer spacings within the bottom trilayer correspond to those of a bulk-truncated CeO_2_ (111) under zero stress, and the in-plane atom positions correspond to those in the corresponding strained CeO_2_ bulk.

To systematically investigate how strain affects the relative stability of isolated surface and subsurface oxygen vacancies in the near-surface of CeO_2_ (111), as well as the formation of vacancy pairs, and the localization of the excess charge, reduced strained slabs were created. Isolated surface and subsurface vacancies with different configurations for the Ce^3+^'s were considered with both 5 × 5 and 2 × 2 periodicities. Vacancies were placed near the surface, i.e., within the outermost trilayer, and the Ce^3+^'s within the (two) outermost cationic plane(s) for the 5 × 5 (2 × 2) periodicity. Moreover, using the larger 5 × 5 surface unit cell, nearest and next-nearest neighbor surface and subsurface vacancy pairs with various configurations for the Ce^3+^'s were considered. In order to obtain distinct configurations of the reduced Ce^3+^ sites, a two-step relaxation procedure was applied. In the first step, we replaced the selected Ce^4+^ with Ce^3+^ ions, i.e., for the latter we used PAW potentials for which the 4*f* ^1^ state was moved to the core, and performed non-spin polarized calculations. The so-obtained relaxed structure was further optimized using the regular Ce^4+^ PAW potentials and spin-DFT. We limit the discussion to high-spin states because the difference between these states and any other spin state is <0.01 eV (Keating et al., 2009). The oxidation state of a given Ce atom has been estimated by considering its local magnetic moment (the difference between up and down spin on the atoms), which can be estimated by integrating the site- and angular momentum-projected spin-resolved density of states over spheres with radii chosen as the Wigner–Seitz radii of the PAW potentials. For reduced Ce ions, the occupation of Ce *f* states is close to one, and the magnetic moment is ~1 μ_B_. Hence, those ions are referred to as Ce^3+^ (4*f* ^1^).

## Results and Discussion

**Figure 1:**
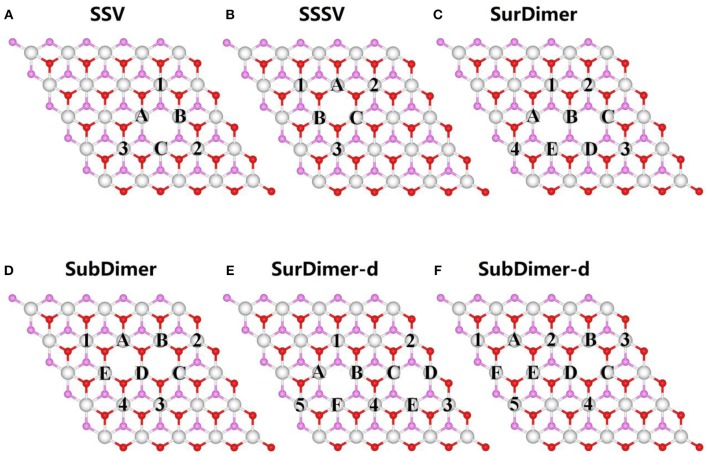
Structural model of the CeO_2_ (111) unit cell with 5 × 5 periodicity. **(A)** single surface vacancy, SSV, **(B)** single subsurface vacancy, SSSV, **(C)** nearest neighbor surface oxygen vacancy dimer, SurDimer, **(D)** nearest neighbor subsurface oxygen vacancy dimer, SubDimer, and **(E)** third-nearest neighbor surface oxygen vacancy pair, SurDimer-d. **(F)** third-nearest neighbor surface oxygen vacancy pair, SubDimer-d. Ce cations in the outermost trilayer are shown as white balls. Surface and subsurface oxygen atoms are shown as red and pink balls, respectively. The labeled cerium atoms denote possible Ce^3+^ locations on nearest-neighbor (A, B, C, D….) or next-nearest-neighbor (1, 2, 3, 4….) sites to the vacancies. Only the three outermost atomic layers are shown for simplicity.

**Figure 2:**
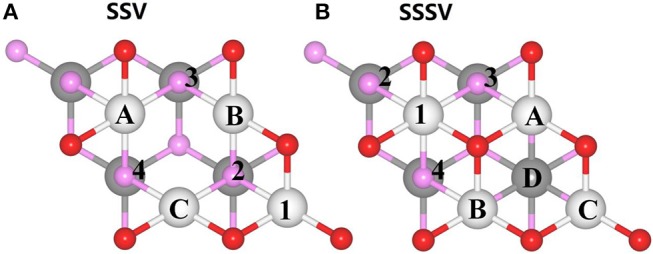
Structural model of the CeO_2_ (111) unit cell with 2×2 periodicity. **(A)** single surface vacancy, SSV, and **(B)** single subsurface vacancy, SSSV. The color scheme for atoms and vacancies corresponds to that used in Figure 1. The labeling scheme for the captions also corresponds to that in Figure 1. Only the five outermost atomic layers are shown for simplicity.

We have modeled the reduced CeO_2_ (111) surface by removing one single surface (SSV) or subsurface (SSSV) vacancy from a supercell with 5 × 5 and 2 × 2 periodicities, as shown in Figures 1, 2, respectively. Nearest (NN) and next-nearest neighbor (NNN) Ce ions to vacancies are labeled with uppercase letters (A, B, C, D, …) and numerals (1, 2, 3, 4, …), respectively. Employing the larger supercell, first-neighbor vacancy dimers at the surface or in the subsurface (SurDimer and SubDimer), as well as corresponding pairs with a vacancy separation corresponding to that of third-nearest neighbors in the oxygen plane (SurDimer-d and SubDimer-d), have also been considered (cf. Figure 2). Stress ranging from −5% (compressive) to +5% (tensile) has been applied to both supercells as mentioned in section Methodology. It is known that under zero applied stress, NNN sites in the outermost cerium layer are the energetically preferred locations of the Ce^3+^ ions, but they would rather be in NNN sites of a deeper layer than next to a vacancy in the outermost one (Murgida and Ganduglia-Pirovano, 2013). Since under applied stress preferences may change, for the strained surfaces with 5 × 5 (Tables S1–S7) or 2 × 2 (Table S8) periodicity, vacancy structures corresponding to the most relevant combinations of the possible locations of the Ce^3+^ ions have been considered.

The averaged vacancy formation energy (*E_f_*) was calculated as:

(1) $$\begin{array}{r} E_{f}=\frac{1}{n}\left[E_{defect}+\frac{n}{2}E_{O_{2}}-E_{perfect}\right] \end{array}$$

where *E_defect_* and *E_perfect_* are the total energies of the (relaxed) defective (reduced) and perfect (clean) slabs, respectively, *n* is the number of oxygen vacancies, and *E*_*O*_2__is the total energy of the isolated O_2_ molecule in its triplet ground state.

### Low Vacancy Concentration

#### Stability, Structural Relaxation, and Excess Charge Localization

**Figure 3:**
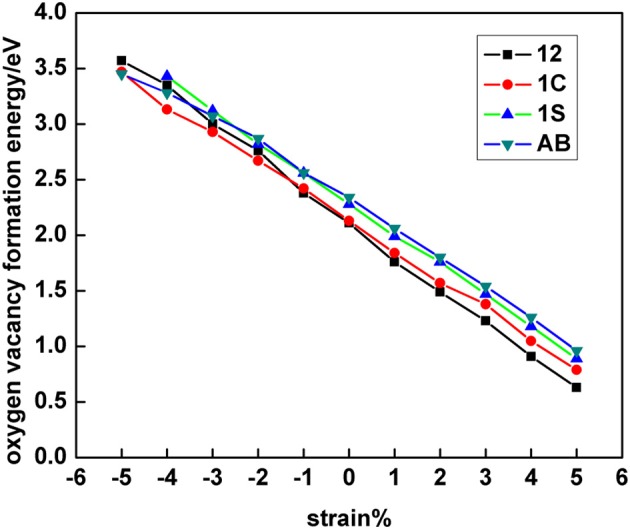
Single surface oxygen vacancy (SSV) formation energy at the 5 × 5 CeO_2_ (111) surface as a function of strain and for distinct polaronic structures, which are labeled according to the location of the two Ce^3+^ as shown in Figure 1.

**Figure 4:**
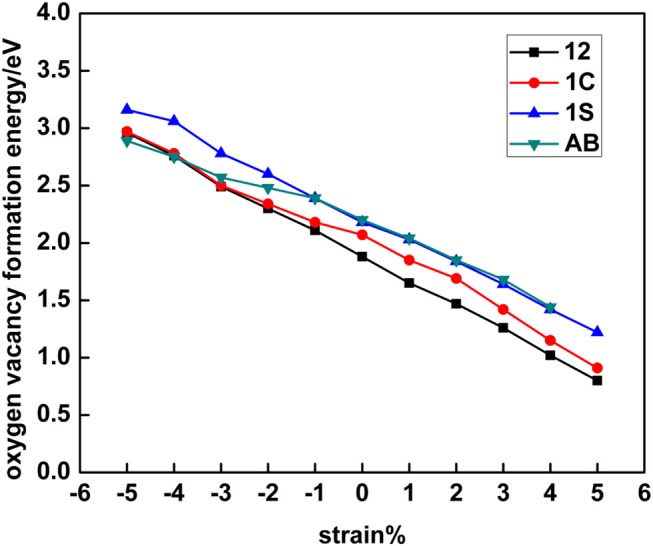
Single subsurface oxygen vacancy (SSSV) formation energy at the 5 × 5 CeO_2_ (111) surface as a function of strain and for distinct polaronic structures, which are labeled according to the location of the two Ce^3+^ as shown in Figure 1.

Figures 3, 4 summarize the calculated vacancy formation energy for all 5 × 5 structures with a SSV and a SSSV vacancy, respectively. For the surface vacancy, configurations with both Ce^3+^ ions in the outermost cationic plane and either in the first coordination shell (AB), in the second (12), or one in each shell (1C) were considered (Figure 1). In addition, a configuration with both Ce^3+^ ions in the second coordination shell, but one in the outermost cationic plane and the other one in the plane beneath, was considered (1S) (Figure S1). Under 0% tension, the 12 configuration is by 0.23 eV more stable than the AB one (Figure 3), in line with previous works (Ganduglia-Pirovano et al., 2009; Li et al., 2009; Murgida and Ganduglia-Pirovano, 2013; Han et al., 2018). As the tensile strain is increased from 0 to +5%, the 12 >1C >1S >AB stability trend does not change although the difference between the most stable configuration (12) and the least stable one (AB) becomes larger (0.33 eV under +5% strain, Table S1). In contrast, if we look at the surface under large compressive strain (≥ −5%), the trend is reversed, i.e., AB >1C>12; (the 1S configuration is not stable); under −5% strain, the configuration with both Ce^3+^ ions in the first coordination shell (AB) is by 0.12 eV more stable than that with the Ce^3+^ ions in the second coordination shell (12).

For the subsurface vacancy, configurations with both Ce^3+^ ions in the outermost cationic plane and either in the first coordination shell (AB) or in the second (12), or one in each shell (1C), as well as with both Ce^3+^ ions in the second coordination shell, but one in the outermost cationic plane and the other one in the plane beneath (1S), were also considered (cf. Figure 1 and Figure S1). The behavior of the SSSV is comparable to that of the SSV, although the slopes of the energy-strain curves for the SSSV are somewhat smaller than those of the SSV (cf. Figures 3, 4). Under 0% tension, the SSSV-12 configuration is by 0.32 eV more stable than the AB one, in line with previous works (Figure 4) (Ganduglia-Pirovano et al., 2009; Li et al., 2009; Murgida and Ganduglia-Pirovano, 2013; Han et al., 2018). The 12 >1C >1S >AB stability trend obtained under 0% tension, does not change upon increasing the tensile strain from 0 to +4% (the AB configuration is not stable under +5% strain), but the difference between the 12 and AB configurations becomes larger, for example, it is 0.42 eV under +4% strain (Table S2). If the surface is compressed, for example by −5%, the most stable configuration has both Ce^3+^ ions in the first coordination shell (AB), and that with the Ce^3+^ ions in the second coordination shell (12), is by 0.07 eV less stable.

**Figure 5:**
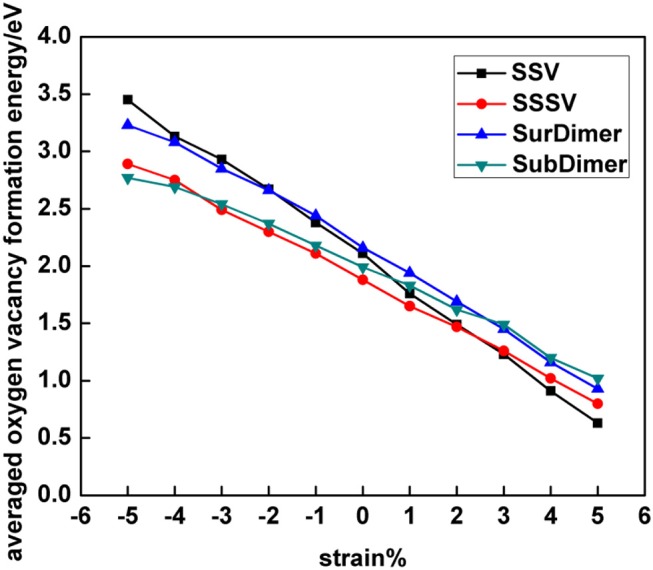
The averaged oxygen vacancy formation energy of the most stable SSV, SSSV, SurDimer, and SubDimer structures with 5 × 5 periodicity as a function of strain.

Before analyzing the origin of the observed transitions between NNN-NNN (12) and NN-NN (AB) Ce^3+^ configurations for both vacancy types under large compressive strain (cf. section Electronic Density of States), we first discuss how their relative stability is influenced by strain. Figure 5 collects the results of the most stable SSV and SSSV configurations for each value of the applied stress (cf. Figures 3, 4, and Tables S1, S2, S7). In agreement with the literature (Ganduglia-Pirovano et al., 2009; Li et al., 2009; Murgida and Ganduglia-Pirovano, 2013), we find that under 0% tension, the subsurface vacancy position is by 0.23 eV more stable than the surface one. Under compressive strain, from 0 to −5%, the SSSV remains more stable than the SSV, but the difference becomes larger as the compression increases, for example under −5% compression, the SSSV is by 0.56 eV more stable than the SSV. However, as the tensile tension increases from +3%, the relative stability reverses and the SSV becomes more stable.

**Figure 6:**
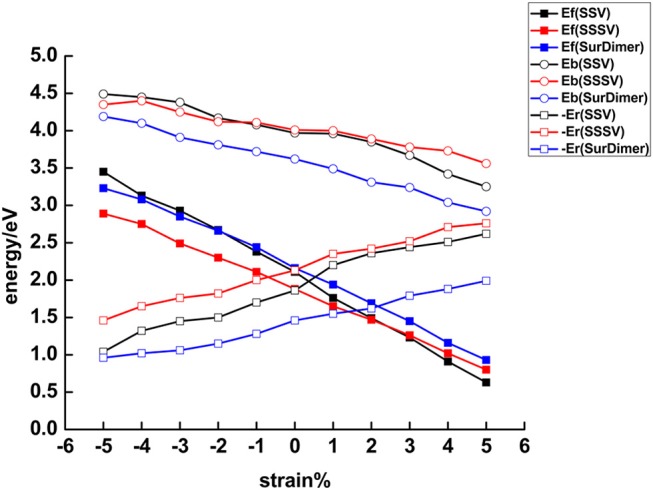
The (average) near-surface oxygen vacancy formation energy, **E_f_* = *E_b_** + **E_r_**, as a function of strain (5 × 5 periodicity). *E_b_* is the energy cost to create a near-surface oxygen vacancy structure without allowing for lattice relaxations, i.e., the bond breaking energy, and *E_r_*, the energy gained from lattice relaxations in the presence of the vacancies.

As vacancy-induced lattice relaxations are known to play an important role in the stabilization of both a particular Ce^3+^ configuration and the subsurface vacancy position in the near-surface of CeO_2_ (111) under 0% strain, (Ganduglia-Pirovano et al., 2009; Li et al., 2009) we analyze their effects on the strained surfaces. To this end, we define two contributions to the vacancy formation energy, *E_f_* = *E_b_* + *E_r_* (cf. Equation 1), namely, the bond breaking energy, *E_b_*, as the energy cost to create a vacancy without allowing for lattice relaxations, i.e., $E_{b}=E_{defect}^{unrelax}+\frac{1}{2}E_{O_{2}}-E_{perfect}$, and the energy gained from the structural relaxation in the presence of the vacancy, *E_r_*, i.e., $E_{r}=E_{defect}^{relax}-E_{defect}^{unrelax}$. In Figure 6 and Table S9, we show how these different contributions depend on strain.

For both SSV and SSSV, *E_b_* is largest for the highest compression and smallest for the largest tensile stress, i.e., the correlation between *E_b_* and strain is negative. To create unrelaxed surface and subsurface oxygen vacancies, three Ce–O_s_ and four Ce–O_ss_ bonds, respectively, have to be cut. For the SSV, the Ce_1_-O_s_ bond lengths (Ce_1_ and O_s_ are in the outermost cationic and anionic layers, respectively) vary from 2.30 to 2.43 Å as the stress changes from −5 to +5% (Table 1), thus, the observed *E_b_* vs. strain trend is in line with the weakening of the bonds upon increasing tensile strain. For the SSSV, the corresponding variations in the three Ce_1_-O_ss_ bond lengths are practically identical to those of the SSV, but the Ce_2_-O_ss_ bond (Ce_2_ and O_ss_ are in the second cationic and anionic layers, respectively) is contracted from 2.52 (−5%) to 2.26 (+5%). In any case, the observed *E_b_* vs. strain trend for the SSSV vacancy is in line with the weakening of the average Ce–O_ss_ bond length upon increasing tensile strain, which varies from 2.35 (−5%) to 2.40 (+5%).

**Table 1:** O–Ce bond lengths (in Å) between a surface oxygen atom at the fully oxidized strained CeO_2_ (111) surface (5 × 5 periodicity), O_s_, and its three nearest neighbor cerium atoms in the outermost cerium layer, Ce_1_, as well as between a subsurface oxygen atom, O_ss_, and its three (one) nearest neighbor cerium atoms in the first (second) cerium layer, Ce_1_ (Ce_2_).

**Strain %**	**−5**	**−4**	**−3**	**−2**	**−1**	**0**	**+1**	**+2**	**+3**	**+4**	**+5**
O_s_−Ce_1_	2.30	2.31	2.32	2.33	2.35	2.36	2.37	2.39	2.40	2.41	2.43
O_ss_−Ce_1_	2.29	2.30	2.32	2.33	2.35	2.36	2.38	2.40	2.41	2.43	2.45
O_ss_−Ce_2_	2.52	2.48	2.45	2.42	2.40	2.37	2.35	2.33	2.31	2.29	2.26

Moreover, we observe that the *E_b_* vs. strain curves for both the SSV and the SSSV resemble straight lines but with a different slope, i.e., the curves cross (Figure 6). Under the largest tensile strain (+5%), three Ce_1_-O_s_ bonds of 2.43 Å for the SSV have to be cut (Table 1), whereas for the SSSV they are three Ce_1_-O_ss_ bonds of 2.45 Å and one particularly strong Ce_2_-O_ss_ bond of 2.26 Å; hence, creating an unrelaxed SSV is preferred. As already mentioned, upon compression the Ce_2_-O_ss_ bond expands in such a way that for the largest compressive strain (−5%), three Ce–O_s_ bonds with an average distance of 2.30 Å and four Ce–O_ss_ ones of 2.35 Å have to be cut to create an unrelaxed SSV and SSSV, respectively (Table 1). The latter is energetically preferred.

In Figure 6 we further compare the energy gained from structural relaxation in the presence of oxygen vacancies, *E_r_* (<0), as a function of surface strain. The *E_r_* vs. strain curves also resemble straight lines with a different slope for the SSV and SSSV, but the curves do not cross within the interval of −5 to +5% strain values. The lattice relaxations are the result of both the presence of the oxygen vacancy, which for example induces the neighboring O (Ce) ions to move toward (away from) the vacancy, and the localization of the excess charge driving the Ce^4+^ → Ce^3+^ reduction of two cations. For isolated oxygen vacancies in bulk ceria under strain (De Souza et al., 2012), it has been shown that the energy gained due to the displacements of the atoms upon relaxation increases almost linearly with the increasing of the tensile stress, independent of the sites on which the excess charge is localized, but the gain is more pronounced when the localization occurs at NNN cationic sites to the vacancy. Similarly, for both SSV and SSSV vacancies in the near-surface of CeO_2_ (111), the energy gained from structural relaxation is smallest for the biggest compressive strain and largest for the highest tensile stress, i.e., the correlation between the absolute value of *E_r_*and strain is positive. We note that the energy gain is always largest for the SSSV within the −5 to +5% strain interval, which is related to the fact that the numbers of neighboring O and Ce ions that will be displaced upon the creation of a SSSV are larger than the corresponding ones for a SSV. Moreover, the relaxation energy gained from the localization of the excess charge also favors the SSSV; for the unrelaxed SSSV and SSV structures, the two excess electrons are equally shared by four and three NN Ce ions, respectively, and thus, the energy gained due to the 4 × Ce^0.5+^ → 2 × Ce^3+^ charge localization in the case of the SSSV, is larger than the corresponding one for the SSV, namely, 3$\times Ce^{0.\hat{6}+}$ → 2 × Ce^3+^. Furthermore, with respect to the above-mentioned smallest gain in the relaxation energy for the largest compressive strain for both SSV and SSSV, we recall here –and address below– that the Ce^3+^ ions in the first coordination sphere are preferred when the surface is substantially compressed and then, it is expected that the contribution associated with the relocation of electrons will be lower when it occurs between first neighbors.

Summarizing, there is an almost linear correlation between both contributions to the oxygen vacancy formation and the surface strain. The correlation between the bond breaking energy, *E_b_*, and strain, is negative and that between the absolute energy gained due to relaxation, –*E_r_*, and strain is positive. The slopes of the almost linear *E_b_* vs. strain and *E_r_* vs. strain relationships are not the same for the SSV than for the SSSV. The preference for the SSSV under compressive strain and for the SSV under tensile strain (>3%) is the result of the different behaviors of the contributions to create the corresponding vacancies with strain. For example, for the −5% compressed surface, both *E_b_* and *E_r_* favor the SSSV by 0.14 and 0.42 eV, respectively, and thus, the SSSV is by 0.56 eV more stable than the SSV. However, for the +5% strained surface, *E_b_* favors the SSV by 0.31 eV and *E_r_* the SSSV by 0.14 eV, and, as a result, the SSV is by 0.17 eV more stable than the SSSV.

#### Electronic Density of States

**Figure 7:**
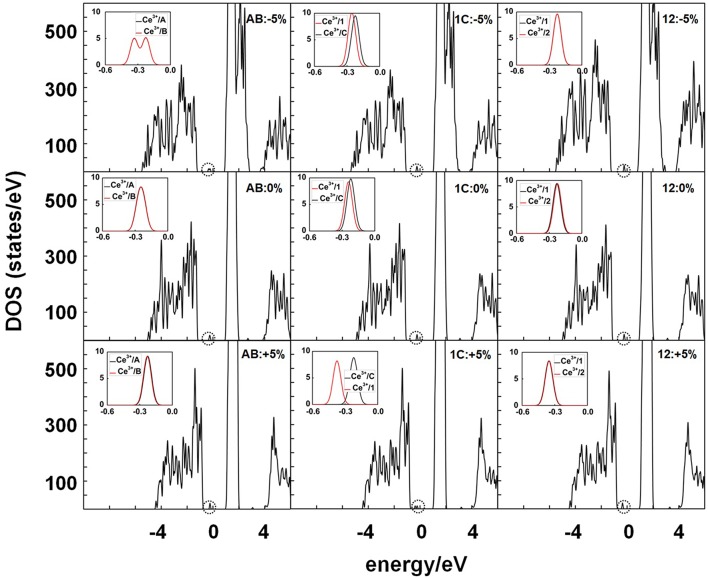
Densities of states (DOS) summed over spin projections and all atoms for a SSV under −5, 0, and +5% strain with different Ce^3+^ configurations, namely, AB, 1C, and 12 and 5 × 5 periodicity. The Fermi level is set as the zero energy value, below which the states are occupied. The occupied Ce 4*f* states are marked with black dashed circles, which are shown magnified in the insets.

The effects of strain on the distribution of the excess charge are also reflected in the calculated densities of states (DOS). In pure CeO_2_, all valence Ce states, including the 4*f* states, are empty and the system is a wide gap insulator with a measured fundamental band gap of 6 eV between the valence and the conduction bands (Pfau and Schierbaum, 1994), which are formed predominantly by the O 2*p* and Ce 5*d* states, respectively. The vacant 4*f* states lie in the O_2p_-Ce_5d_ gap. PBE+*U* underestimates the O_2p_-Ce_5d_ gap of bulk CeO_2_ by ~12% (5.3 eV) (Da Silva et al., 2007). The formation of a pair of Ce^3+^ ions following the creation of an oxygen vacancy results in the appearance of split-off (defect) states of the initially empty Ce 4*f* band, lying inside the O_2p_-Ce_5d_ gap below the Fermi level (Jerratsch et al., 2011). If the two Ce^3+^ ions occupy different cationic shells around the vacancy, the resulting variation in the chemical environment leads to a splitting of the two filled Ce^3+^ *f* levels, which could be detected with STM spectroscopy (Jerratsch et al., 2011). The densities of states for the example of an SSV at the CeO_2_ (111) surface with the Ce^3+^s in either the 12, 1C, or AB configuration under −5, 0, or +5% strain (Figure 7) are discussed in the following.

In the 12 configuration, the two next-nearest neighbor Ce^3+^ ions have equal coordination number (CN = 7) and filled *f* orbitals with the same energy as they experience the same chemical environment, and hence no splitting of the *f* orbitals is expected; this is certainly observed for the 12 configurations within the −5 to +5% strain range (Figure 7; Table S10). Similarly, for the AB configuration with the two Ce^3+^ ions in nearest neighbor sites, and experiencing the same chemical environment (CN = 6), no splitting of the *f* orbitals is expected. However, two peaks are observed in the DOS for −5% compression with a splitting of the order of 0.16 eV. The reason for the splitting is not that the Ce^3+^s are located in different environments but a hybridization of the polaronic states, which causes a spread of charges across both adjacent sites. That is, the polarons are no longer fully localized in one specific Ce site, rather, the polaronic charge is shared between the two sites in a sort of bonding/anti-bonding configuration (cf. isosurfaces in Figure S2). Finally, for the 1C configuration with one nearest and one next-nearest Ce^3+^ (CN = 6 and 7, respectively), we find a splitting of the *f* orbitals of the order of 0.1 eV for −5 and 0% strain, but increases to about 0.2 eV for +5% tensile strain (Table S10). Before discussing the changes in the positions of the occupied *f* states in the O_2p_-Ce_5d_ gap as a consequence of in-plane strain here below, we note that both compressive and tensile strain decrease the O_2p_-Ce_5d_ gap (Figure 7; Table S10), and that the width of the valence band widens (shrinks) as the lattice is compressed (expanded).

**Figure 8:**
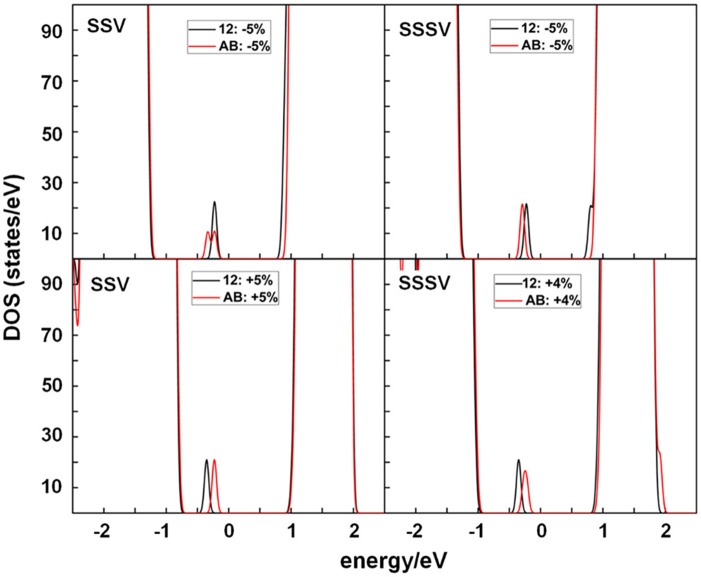
Densities of states (DOS) summed over spin projections and all atoms for a SSV and SSSV under −5%, and +5%, or +4% strain with AB and 12 Ce^3+^ configurations and 5 × 5 periodicity. The Fermi level is set as the zero energy value, below which the states are occupied.

The position of the occupied *f* states in the O_2p_-Ce_5d_ gap is hereby given as O_2p_-Ce_4f_, i.e., the difference between the eigenvalue of the lowest occupied *f* state and the top of the valence band. We note that, as the in-plane strain changes from −5 to +5%, the occupied *f* states are shifted toward the valence band (Table S10), but the amount by which they are shifted depends on the actual positions of the Ce^3+^ with respect to the vacant site. Figure 8 compares the total DOS of the strained SSV and SSSV with the AB and 12 configurations and shows that under surface compression, the characteristic polaron peaks of the AB configuration lie lower in energy than those of the 12 one, whereas under surface expansion, the situation is reversed. This is in line with the above observed changes in the relative stability of the NN-NN (AB) and NNN-NNN (12) Ce^3+^ configurations for both SSV and SSSV vacancy types (cf. Figures 3, 4) under varying strain, i.e., AB configurations are stable under large compressive strain whereas 12 ones are stable otherwise.

### High Vacancy Concentration

**Figure 9:**
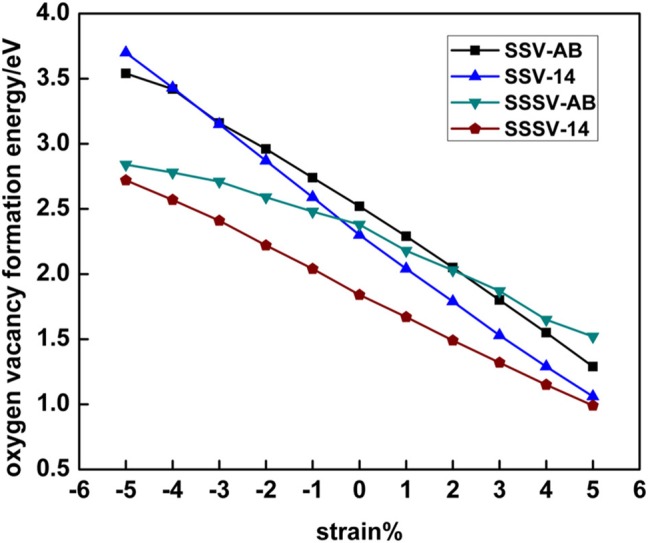
Oxygen vacancy formation energy for single surface (SSV) and subsurface (SSSV) vacancies at the 2 × 2 CeO_2_ (111) surface as a function of strain and for distinct polaronic structures, which are labeled according to the location of the two Ce^3+^ as shown in Figure 2.

In this section we analyze the results for a SSV and a SSSV in the near-surface of CeO_2_ (111) under strain with 2 × 2 periodicity (Figure 9; Table S8), and compare them to those obtained with the 5 × 5 unit cell. In the 2 × 2 unit cell under zero applied stress, the most stable SSV and SSSV configurations have both Ce^3+^ ions in NNN sites, one in the outermost cationic layer, and the other in the layer beneath, i.e., both configurations are labeled as 14 (cf. Figure 2), in line with previous calculations (Murgida and Ganduglia-Pirovano, 2013). These SSV (14) and the SSSV (14) configurations are by 0.22 and 0.54 eV more stable, respectively, than the corresponding ones with the Ce^3+^ ions in NN sites (AB). We note that at the lower vacancy concentration (5 × 5 unit cell) and zero applied stress, the SSV and the SSSV configurations with both Ce^3+^ ions in the second coordination shell (12) are by 0.23 and 0.32 eV more stable, respectively, than the corresponding ones with Ce^3+^ ions in the first coordination shell (AB). For the SSV and SSSV oxygen vacancies with 2 × 2 periodicity under 0% strain, the 14 >1B >AB and 14 > 1A ≈ 1D > AD ≈ AB stability trends, respectively, are obtained (Table S8) (Ganduglia-Pirovano et al., 2009; Murgida and Ganduglia-Pirovano, 2013). As the tensile strain is increased from 0 to +5%, the SSV- and SSSV-14 configurations remain more stable with respect to the corresponding AB ones. For example, for the +5% stretched surface, the SSV- and SSSV-14 configurations are by 0.23 and 0.53 eV more stable, respectively, than the corresponding SSV- and SSSV-AB ones (Figure 9; Table S8). However, in line with the results for the lower vacancy concentration (5 × 5 unit cell), for both vacancy types under compressive strain, an increasing preference for configurations with both Ce^3+^ ions in the first coordination shell (AB) is observed. For example, for −5% strain, the SSV-AB configuration is by 0.16 eV more stable than the SSV-14 (Figure 9; Table S8); for the SSSV, an even larger compressive strain would be needed to observe the crossing between the AB and 14 configurations (cf. Figure 9), but the tendency is clear. In summary, for both vacancy concentrations, the ground-state configuration of both vacancy types varies whether tensile or compressive stress is applied, namely, under tensile strain, both Ce^3+^ ions do *not* prefer the first coordination sphere of the vacancy, but under large compressive strain they do.

With respect to the relative stability between the SSV and SSSV when stress is applied and the concentration of vacancies is larger (Figure 9), a behavior very similar to that already discussed for the case of low concentration is observed (cf. Figure S3, Figure 5). In agreement with the literature (Ganduglia-Pirovano et al., 2009; Murgida and Ganduglia-Pirovano, 2013), we find that under 0% tension, the subsurface vacancy position is by 0.46 eV more stable than the surface one; at the lower vacancy concentration the difference is by a factor of two smaller. Under compressive strain, from 0 to −5%, the SSSV remains more stable than the SSV, but the difference becomes larger as the compression increases, for example, under −5% compression, the SSSV is by 0.82 eV more stable than the SSV. However, under tensile strain, the larger relative stability of the SSSV as compared to the SSV becomes smaller and smaller as the strain increases from 0 to +5%, but an even larger tensile strain would be needed to observe the crossing between the corresponding *E_f_* vs. strain curves; at the lower vacancy concentration the crossover occurs at about +3% strain (cf. Figure 6). For the higher vacancy concentration, we have also evaluated the bond breaking, *E_b_*, and the relaxation energy, *E_r_*, contributions to the vacancies formation energies as a function of surface strain (Figure S3; Table S11), which behave similarly to the case of the lower vacancy concentration discussed above. Both contributions resemble straight lines with a different slope for the SSV and SSSV, and the correlation between *E_b_* and strain is negative, whereas that between –*E_r_* and strain is positive. For the −5% compressed surface, both *E_b_* and *E_r_* favor the SSSV by 0.11 and 0.71 eV, respectively, and thus, the SSSV is by 0.82 eV more stable than the SSV. However, for the +5% strained surface, *E_b_* favors the SSV by 0.14 eV and *E_r_* the SSSV by 0.21 eV, and, as a result, the SSSV is only by 0.07 eV more stable than the SSV.

### Vacancy Clustering

As mentioned above (section Introduction), under zero applied stress, isolated subsurface vacancies are more stable than surface ones, and the interaction between nearest neighbor vacancies at the surface or in the subsurface is repulsive. We here consider strained reduced surfaces with either first-neighbor vacancy dimers at the surface (SurDimer), or in the subsurface (SubDimer), or pairs with a vacancy separation corresponding to that of third-nearest neighbors in the surface oxygen plane (SurDimer-d), or in the subsurface one (SubDimer-d), cf. Figure 1. Due to the large number of possible different configurations of the four Ce^3+^ created upon formation of a vacancy dimer, we have selected a set that mostly involve combinations of nearest-neighbor (A, B, C, D….) or next-nearest-neighbor (1, 2, 3, 4….) sites to the vacancies (Tables S3–S6). Figure 5 shows the averaged vacancy formation energy of the most stable structures as a function of strain (Table S7). As expected (Murgida and Ganduglia-Pirovano, 2013; Han et al., 2018), under zero applied stress, the interaction between first-neighbor surface and subsurface vacancies is repulsive; creating two well-separated surface and subsurface vacancies is energetically more favorable by about 0.1 [Δ*E_f_* = 2 *E_f_* (Surdimer)– 2 *E_f_* (SSV), cf. Equation 1] and 0.2 eV [Δ*E_f_* = 2 *E_f_* (Subdimer)– 2 *E_f_* (SSSV)], respectively, than the corresponding pair formation. Tensile strain not only does not help neither the formation of surface nor subsurface vacancy pairs, but makes the preference for having corresponding isolated species even more pronounced, for example, for +5% tensile strain, by about 0.6 and 0.4 eV, respectively. However, upon lattice compression, the SurDimer and the SubDimer become more stable than the corresponding isolated species, for example, for −5% compressive strain, by about 0.4 and 0.2 eV, respectively, with the SubDimer being by about 0.9 eV more stable than the SurDimer.

In order to understand why compression favors vacancy clustering, for the example of the SurDimer, we have analyzed the two contributions to the vacancy dimer formation energy, namely, the bond breaking energy, *E_b_*, and the energy gained from the structural relaxation in the presence of the vacancies, *E_r_*, and compared them to those of the SSV (Figure 6; Table S9). Figure 6 shows that within the −5 to +5% strain interval, creating an unrelaxed SurDimer is always energetically preferred over having two isolated SSV species, for example by 0.7 [Δ*E_b_* = 2 *E_b_* (Surdimer)– 2 *E_b_*(SSV)] and 0.6 eV under 0 and −5% strain, respectively (Table S9). The reason for this is likely due to the way the four excess electrons are “delocalized” among the nearest-neighbor cations in the unrelaxed structures, i.e., for the SurDimer, the shared cation (B, Figure 1) is fully reduced to Ce^3+^, and the other four (A, C, E, and D), are partially reduced to Ce^0.75+^, whereas in the case of two isolated SSV, the three nearest-neighbor cations of each vacancy would share two excess electrons (3$\times Ce^{0.\hat{6}\;+}$).

With respect to the energy gained from lattice relaxations, it is always smaller for the SurDimer as compared to two isolated SSV, expectedly, but the difference becomes smaller and smaller as the strain decreases from +5 to −5% (Table S9). For instance, under 0% (−5%) strain, the gain amounts to 1.46 (0.96) and 1.86 (1.04) eV/vacancy for the SurDimer and a SSV, respectively. Consequently, under 0% (−5%) strain, Δ*E_f_* = Δ*E_b_* + Δ*E_r_* = −0.7 + 0.8 (−0.6 + 0.16) = +0.1 (−0.44), i.e., it is only when the energy gained from lattice relaxations is considered that the preference for dimer formation under compressive strain (>−2%) can be explained.

We finally note that as for the case of the isolated species discussed above, for which compressive (tensile) strain favors NN (NNN) Ce^3+^ configurations, for the dimeric species, a similar tendency is observed, for example, the stable SurDimer and SubDimer configurations under −5% compressive strain have all four Ce^3+^ ions in the first cationic coordination shell of the vacancies (Tables S3, S4).

## Conclusion

This work shows the important effect of lattice strain on the relative stability of different types of near-surface oxygen vacancies at the CeO_2_ (111) surface, and on their ground-state energy configurations with respect to the localization of the excess charge. We have found three situations of energy crossover: (*i*) isolated surface vs. sub-surface oxygen vacancy species; (*ii*) Ce^3+^ within the first vs. second coordination shell of the vacancies; (*iii*) isolated species vs. first-neighbor vacancy dimers. Under 0% strain, isolated subsurface oxygen vacancies with both Ce^3+^ ions within the second coordination shell of the vacancies are favored, and the interaction between first-neighbor vacancies is repulsive. However, if a large tensile stress is applied, isolated surface vacancies with both Ce^3+^ in the second coordination shell become more stable than the subsurface ones, with both Ce^3+^ in next-nearest neighbor sites too. Moreover, if a large compressive strain is applied, isolated subsurface vacancies are more stable than surface ones, as in the case of 0% strain, but for both vacancy types configurations with both Ce^3+^ in the first coordination shell of the vacancies are favored over those with both Ce^3+^ in the second coordination shell. Finally, under large compressive strain, the formation of first-neighbor vacancy dimers at the surface and in the subsurface, with the four Ce^3+^ in nearest-neighbor sites, is more stable than having the two corresponding isolated species, with the subsurface vacancy dimer being more stable than the surface one.

We have further analyzed the effects of lattice strain on the energy cost to create a single vacancy without allowing for lattice relaxations, as well as on the energy gained due to lattice relaxations, and found that the preference for the surface or subsurface sites is the result of the different dependences of these energies on the applied stress. Regarding the Ce^3+^ switched preference in favor of the first coordination shell of the vacancies under compressive strain, we have inspected the calculated densities of states and found that the Ce^3+^ switched preference is related to the exchange in the relative positions of highest occupied Ce^3+^ *f* states in the O_2p_-Ce_5d_ gap region.

Generally speaking, the vacancy structure at ceria surfaces is of importance for technological applications. Clearly, the finding that the relative stabilities of vacancy structures do depend on the applied in-plane strain, particularly in relation to the formation of vacancy aggregates, cannot be ignored when considering that in most applications ceria is employed within a heterostructure or as a supported thin film in which ceria can be strained.

## Data Availability

The raw data supporting the conclusions of this manuscript will be made available by the authors, without undue reservation, to any qualified researcher.

## Author Contributions

MVG-P and YG supervised the whole project. Z-KH and LZ performed the calculations. Z-KH, LZ, MVG-P, and YG wrote the paper. All authors participate in the discussions.

### Conflict of Interest Statement

The authors declare that the research was conducted in the absence of any commercial or financial relationships that could be construed as a potential conflict of interest.
