# Corrigendum: The Structure of Oxygen Vacancies in the Near-Surface of Reduced CeO_2_ (111) Under Strain

**DOI:** 10.3389/fchem.2019.00795

**Published:** 2019-11-22

**Authors:** Zhong-Kang Han, Lei Zhang, Meilin Liu, Maria Verónica Ganduglia-Pirovano, Yi Gao

**Affiliations:** ^1^Shanghai Institute of Applied Physics, Chinese Academy of Sciences, Shanghai, China; ^2^Center for Innovative Fuel Cell and Battery Technologies, School of Materials Science and Engineering, Georgia Institute of Technology, Atlanta, GA, United States; ^3^Institute of Catalysis and Petrochemistry, Spanish National Research Council (CSIC), Madrid, Spain; ^4^Zhangjiang Laboratory, Shanghai Advanced Research Institute, Chinese Academy of Sciences, Shanghai, China

**Keywords:** CeO_2_, density functional theory, oxygen vacancy, strain, surface structures

In the original article, there was a mistake in Figure 8 as published. The SSV and SSSV labeling in the top-right and bottom-left panels were interchanged. Moreover, in the bottom-right panel, the Densities of states (DOS) summed over spin projections and all atoms for a SSSV shown, did not correspond to those of the structure under the +4% strain with AB and 12 Ce^3+^ configurations and 5 × 5 periodicity, as indicated in the caption. The corrected Figure 8 appears below.

**Figure 8:**
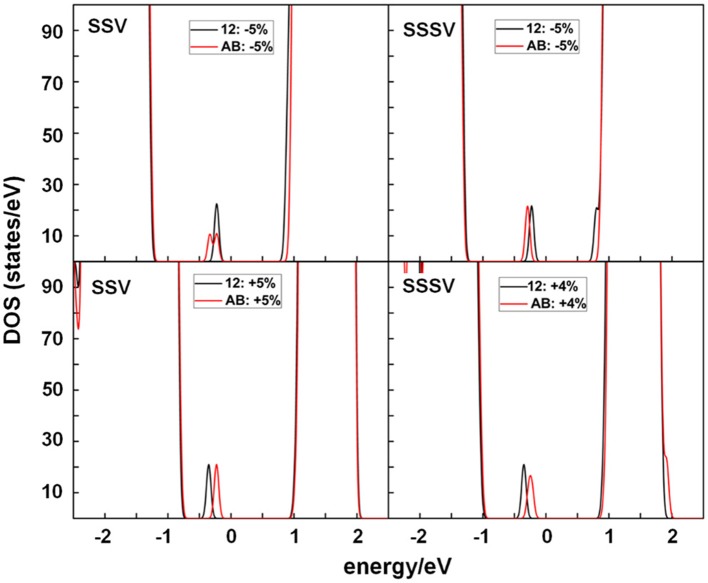
Densities of states (DOS) summed over spin projections and all atoms for a SSV and SSSV under −5%, and +5%, or +4% strain with AB and 12 Ce^3+^ configurations and 5 × 5 periodicity. The Fermi level is set as the zero energy value, below which the states are occupied.

The authors apologize for this error and state that this does not change the scientific conclusions of the article in any way. The original article has been updated.

