# Supplementary material for: The Structure of Oxygen Vacancies in the Near-Surface of Reduced CeO2 (111) Under Strain
Source: Front Chem. 2019 Jun 18;7:436. doi: 10.3389/fchem.2019.00436 (PMC6592146; doi:10.3389/fchem.2019.00436)
Supplement: Supplementary file 1 [file Presentation_1.pdf]

The Near-surface Oxygen Vacancy Structure at the Reduced CeO_2 (111) Surface under Strain

Zhong-Kang Han,[1#†] Lei Zhang,[2] Meilin Liu,[2] M. Verónica Ganduglia-Pirovano[3,*], Yi Gao[1,4*]

[1]Shanghai Institute of Applied Physics, Chinese Academy of Sciences, 201800, Shanghai, P. R. China

[2]Center for Innovative Fuel Cell and Battery Technologies, School of Materials Science and Engineering, Georgia Institute of Technology, Atlanta 30332, GA, USA

[3]Instituto de Catálisis y Petroleoquímica of the Consejo Superior de Investigaciones Científicas, 28049, Madrid, Spain

[4]Shanghai Advanced Research Institute, Chinese Academy of Sciences, 201210，Shanghai, P.R. China

[†]present address: Fritz Haber Institute of the Max Planck Society, Faradayweg 4-6, 14195 Berlin, Germany

Email: vgp@icp.csic.es, gaoyi@sinap.ac.cn

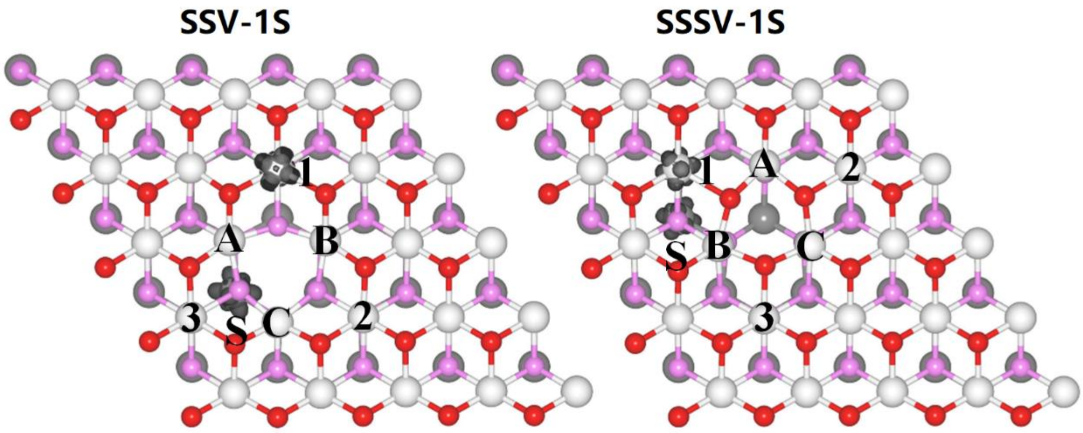

Figure S1. Isosurfaces of the spin density (high spin) for defective $CeO_2(111)$ surfaces with 5×5 periodicity. (a): single surface vacancy, SSV. (b): single subsurface vacancy, SSSV. Ce cations in the outermost trilayer are shown as white balls. Surface and subsurface oxygen atoms are shown as red and pink balls, respectively. The labelled cerium atoms denote possible Ce^{3+} locations on nearest-neighbor (A, B, C, D….) or next-nearest-neighbor (1, 2, 3, 4….) sites to the vacancies in the outermost cationic layer; S denotes a Ce^{3+} in the layer beneath. Only the three outermost atomic layers are shown for simplicity.

Table S1. Oxygen vacancy formation energies (eV) of a SSV on the reduced $CeO_2(111)$ surface with 5×5 periodicity for different configurations of the Ce^{3+} ions. Nearest (NN) and next-nearest neighbor (NNN) Ce ions to vacancies in the outermost cationic layer are labelled with uppercase letters (A, B, C, D, …) and numerals (1, 2, 3, 4, …), respectively; S denotes a Ce^{3+} in the layer beneath.

Strain%	12	1C	1S	AB
+5	0.63	0.79	0.89	0.96
+4	0.91	1.05	1.18	1.26
+3	1.23	1.38	1.47	1.54
+2	1.49	1.57	1.76	1.80
+1	1.76	1.84	1.99	2.06
0	2.11	2.13	2.28	2.34
−1	2.38	2.42	2.56	2.56

−2	2.76	2.67	2.82	2.87
−3	3.00	2.93	3.12	3.07
−4	3.35	3.13	3.43	3.28
−5	3.57	3.47	*	3.45

*configuration changed during geometry optimization.

Table S2. Oxygen vacancy formation energies (eV) of a SSSV in the near-surface of reduced $CeO_2(111)$ with 5×5 periodicity for different configurations of the Ce^{3+} ions. The labelling is the same as in Table S1.

Strain%	12	1C	1S	AB
+5	0.80	0.91	1.22	*
+4	1.02	1.15	1.42	1.44
+3	1.26	1.42	1.64	1.68
+2	1.47	1.69	1.84	1.85
+1	1.65	1.85	2.03	2.04
0	1.88	2.07	2.18	2.20
−1	2.11	2.18	2.39	2.39
−2	2.30	2.34	2.60	2.48
−3	2.49	2.50	2.78	2.57
−4	2.76	2.78	3.06	2.75
−5	2.96	2.97	3.16	2.89

*configuration changed during geometry optimization.

Table S3. Averaged oxygen vacancy formation energies (eV) of a SurDimer on the reduced $CeO_2(111)$ with 5×5 periodicity surface for different configurations of the Ce^{3+} ions. The labelling is the same as in Table S1.

Strain %	Configuration<br>Energy/eV										
+5	24-BE	134-B	12-DE	14-BD	1-BDE	4-ACD	124-D	ABCE	1234	34-BE	24-BS
	0.93	0.93	0.94	0.95	0.99	1.02	1.05	1.07	1.13	1.15	*
+4	1234	134-B	124-E	24-BD	12-DE	14-CD	34-AB	234-S	1-BDE	4-ACD	ACDE
	1.16	1.18	1.19	1.21	1.22	1.23	1.25	1.27	1.32	1.32	1.36
+3	134-B	124-D	1234	34-BE	12-DE	24-BD	14-CD	134-S	4-ACD	1-BCE	ABCD
	1.45	1.46	1.47	1.48	1.49	1.50	1.51	1.54	1.58	1.62	1.65
+2	124-D	12-DE	134-B	1234	34-BE	24-BD	14-BD	124-S	4-BCD	1-BDE	ABCE
	1.69	1.70	1.71	1.71	1.72	1.73	1.74	1.76	1.79	1.81	1.85
+1	134-B	124-S	124-D	12-DE	24-BD	34-BE	14-BD	1234	4-BCD	1-BCE	ABCD
	1.94	1.94	1.96	1.96	1.97	1.97	1.99	1.99	2.01	2.06	2.13
0	24-BD	12-DE	14-BD	4-BCD	134-B	124-D	4-BCD	ABCE	1-BDE	1234	24-SS
	2.16	2.17	2.18	2.19	2.21	2.21	2.22	2.24	2.25	2.30	2.35
−1	134-B	4-BCD	12-DE	14-BD	134-S	24-BE	124-D	34-AB	1-BCE	ABCE	1234
	2.44	2.45	2.46	2.47	2.48	2.49	2.50	2.51	2.51	2.51	2.55
−2	24-BD	4-BCD	134-B	12-DE	14-BD	34-BE	124-D	ABCE	1-BDE	1234	24-SS
	2.66	2.67	2.68	2.68	2.69	2.69	2.70	2.71	2.73	2.77	2.83
−3	24-BD	4-BCE	34-BE	ABCE	12-DE	14-BD	124-D	134-B	1-BDE	1234	24-SS
	2.85	2.86	2.86	2.87	2.88	2.89	2.90	2.91	2.91	2.99	*
−4	ABCD	24-BD	34-BE	12-DE	1-BDE	4-BCE	124-D	134-B	14-BC	1234	ABSS
	3.08	3.09	3.11	3.13	3.13	3.15	3.16	3.16	3.17	3.30	3.32
−5	ABCE	34-BE	1-BDE	14-BD	134-B	12-DE	124-D	1234	ABSS	24-BD	4-BCE
	3.23	3.29	3.30	3.31	3.36	3.36	3.39	3.50	3.51	*	*

*configuration changed during geometry optimization.

Table S4. Averaged oxygen vacancy formation energies (eV) of a SubDimer in the near-surface of reduced $CeO_2(111)$ with 5×5 periodicity for different configurations of the Ce^{3+} ions. The labelling is the same as in Table S1.

Strain %	Configuration<br>Energy/eV										
+5	124-C	1234	34-CE	12-BD	234-A	124-S	ACDE	1-BCD	13-BD	14-AB	4-ABD
	1.02	1.04	1.08	1.09	1.09	1.11	1.54	*	*	*	*
+4	124-C	1234	234-S	34-CE	12-BD	BCDS	124-S	1-BCD	4-ABD	13-BD	14-AB
	1.20	1.24	1.27	1.27	1.31	1.45	1.33	1.46	1.48	*	*
+3	12-DS	12-CD	234-A	BDES	13-BD	1234	14-AB	1-BCD	124-S	4-ABS	34-AD
	1.49	1.51	1.51	1.52	1.52	1.53	1.54	1.54	1.55	1.58	1.65
+2	124-D	12-AD	234-A	1234	34-AB	13-BD	124-S	14-AB	1-BDS	4-BSS	BCDE
	1.62	1.65	1.68	1.69	1.70	1.71	1.73	1.73	1.79	1.80	1.93

+1	14-DS	14-BD	124-D	12-DS	234-A	13-BD	1234	34-AS	1-BDS	4-ABD	BDES
	1.83	1.85	1.86	1.88	1.89	1.92	1.92	1.92	1.99	2.05	2.07
0	124-D	12-AD	13-BD	134-B	1234	34-AB	1-BDS	14-BD	4-ABC	BDES	124-S
	1.99	2.01	2.01	2.02	2.04	2.06	2.08	2.08	2.09	2.10	2.11
−1	124-D	13-BD	234-A	34-AB	12-BD	1-BCD	1234	14-AB	124-S	3-ABD	BDSS
	2.18	2.20	2.28	2.29	2.30	2.30	2.32	2.33	2.33	2.35	2.46
−2	124-D	14-BD	13-BD	12-AD	1-BCD	34-AB	3-ABD	ABDS	1234	124-S	234-D
	2.37	2.39	2.39	2.42	2.43	2.47	2.48	2.53	2.53	2.53	2.55
−3	124-D	14-BD	BCDE	13-BD	1-BCD	12-CD	3-ABD	34-AD	234-D	124-S	1234
	2.54	2.56	2.57	2.57	2.57	2.59	2.64	2.66	2.69	2.71	2.73
−4	13-BD	1-ACD	12-AD	BCDE	124-D	14-BD	3-ABD	34-AB	13-BS	234-D	1234
	2.69	2.71	2.72	2.72	2.75	2.76	2.79	2.80	2.86	2.86	2.94
−5	ACDE	1-BCD	13-BD	ACDS	14-BD	3-ABD	12-DE	124-D	34-AB	234-D	1234
	2.77	2.81	2.83	2.84	2.84	2.86	2.86	2.87	2.96	3.00	3.07

*configuration changed during geometry optimization.

Table S5. Averaged oxygen vacancy formation energies (eV) of a SurDimer-d on the reduced $CeO_2(111)$ with 5×5 periodicity surface for different configurations of the Ce^{3+} ions. The labelling is the same as in Table S1.

Strain %	Configuration Energy/eV									
+5	1234	234-B	134-E	124-F	123-F	24-AE	123-S	12-EF	23-BF	34-BC
	0.74	0.79	0.85	0.85	0.87	0.92	0.93	0.93	0.93	0.94
	4-ABD	13-CF	3-BCF	2-ACE	ABDE					
	0.95	0.96	0.97	1.07	1.12					
+4	1234	234-B	134-E	124-F	24-AE	123-F	13-CF	123-S	34-BC	23-BF
	1.03	1.08	1.13	1.15	1.16	1.17	1.18	1.19	1.20	1.22
	12-EF	4-ABD	3-BCF	2-ACE	ABDE					
	1.22	1.23	1.27	1.31	1.41					
0	1234	234-B	34-BC	134-E	124-F	2-ACE	24-AE	13-CF	123-F	12-EF
	2.15	2.18	2.18	2.19	2.23	2.24	2.24	2.24	2.26	2.29
	4-ABD	23-BF	3-BCF	123-S	ABDE					
	2.30	2.31	2.34	2.35	2.36					
−4	13-CF	3-BCF	234-B	4-ABD	23-BF	124-F	1234	2-ACE	ABDE	123-F
	3.26	3.27	3.28	3.29	3.31	3.32	3.35	3.35	3.36	3.36
	34-BC	12-EF	134-E	24-AE	13-FS					
	3.37	3.39	3.41	*	*					
−5	ABDE	1234	2-ACE	234-B	ABDS	124-F	12-EF	123-F	134-E	13-CF
	3.51	3.56	3.62	3.63	3.64	3.65	3.65	3.65	3.66	*
	23-BF	24-AE	34-BC	3-BCF	4-ABD					
	*	*	*	*	*					

*configuration changed during geometry optimization.

Table S6. Averaged oxygen vacancy formation energies (eV) of a SubDimer-d in the near-surface of reduced $CeO_2(111)$ with 5×5 periodicity for different configurations of the Ce^{3+} ions. The labelling is the same as in Table S1.

Strain %	Configuration Energy/eV									
+5	1245	245-B	14-BE	125-D	145-B	25-AC	124-A	45-AB	125-S	12-DE
	0.83	0.90	0.91	0.91	0.94	0.95	0.99	1.00	1.04	1.11
	15-BD	1-BDE	2-CDF	5-ACE	ACDF					
	*	*	*	*	*					
+4	1245	125-C	245-S	124-S	25-AC	145-B	14-BE	45-BS	12-SS	125-S
	1.02	1.09	1.09	1.12	1.16	1.17	1.21	1.22	1.23	1.24
	15-SS	1-BDE	2-CDF	5-ACE	ACDF					
	1.26	1.36	*	*	*					
0	1245	245-B	14-BE	125-D	124-A	12-DE	2-CDF	125-S	145-B	25-AC
	1.85	1.86	1.88	1.88	1.89	1.91	1.93	1.94	1.94	1.96
	15-BD	5-ACE	1-DES	45-AS	ACDF					
	1.99	2.03	2.08	2.10	2.11					
−4	25-AC	125-D	245-B	45-AB	25-CS	1-BDE	15-BD	12-DE	145-B	ACDF
	2.69	2.71	2.72	2.72	2.73	2.74	2.76	2.76	2.76	2.78
	124-A	5-ACE	2-CDF	14-BE	1245					
	2.78	2.78	2.79	2.79	2.80					
−5	25-AC	14-BE	25-CS	125-D	12-DE	5-ACE	1245	ACDF	1-BDE	245-B
	2.84	2.85	2.88	2.89	2.89	2.89	2.90	2.91	2.91	2.92
	15-BD	45-AB	145-B	2-CDF	124-A					
	2.93	2.94	2.94	2.94	2.95					

*configuration changed during geometry optimization.

Table S7. Averaged oxygen vacancy formation energies (eV) of the most stable SSV, SSSV, SurDimer, SubDimer and SubDimer-d in the near-surface of reduced $CeO_2(111)$ with 5×5 periodicity.

Strain%	SSV	SSSV	SurDimer	Subdimer	SurDimer-d	SubDimer-d
+5	0.63	0.80	0.93	1.02	0.74	0.83
+4	0.91	1.02	1.16	1.20	1.03	1.02
+3	1.23	1.26	1.45	1.49		
+2	1.49	1.47	1.69	1.62		
+1	1.76	1.65	1.94	1.83		

0	2.11	1.88	2.16	1.99	2.15	1.86
−1	2.38	2.11	2.44	2.18		
−2	2.67	2.30	2.66	2.37		
−3	2.93	2.49	2.85	2.54		
−4	3.13	2.75	3.08	2.69	3.26	2.69
−5	3.45	2.89	3.23	2.77	3.51	2.84

Table S8. Oxygen vacancy formation energies (eV) of a SSV and a SSSV at the reduced $CeO_2(111)$ surface with 2×2 periodicity for different configurations of the Ce^{3+} ions. Nearest (NN) and next-nearest neighbor (NNN) Ce ions to vacancies in the outermost cationic layer are labelled with uppercase letters (A, B, C, D, …) and numerals (1, 2, 3, 4, …), respectively.

Strain%	SSV-AB	SSV-1B	SSV-14	SSSV-AB	SSSV-AD	SSSV-1A	SSSV-1D	SSSV-14
+5	1.29	1.07	1.06	1.52	1.06	1.02	0.96	0.99
+4	1.55		1.29	1.65				1.15
+3	1.80		1.53	1.87				1.32
+2	2.05		1.79	2.03				1.49
+1	2.29		2.04	2.18				1.67
0	2.52	2.35	2.30	2.38	2.41	2.00	2.11	1.84
−1	2.74		2.59	2.48				2.04
−2	2.96		2.87	2.59				2.22
−3	3.16		3.15	2.71				2.41
−4	3.42		3.43	2.78				2.57
−5	3.54	3.54	3.70	2.84	3.23	2.85	3.12	2.72

Table S9. Averaged oxygen vacancy formation energies (E_f), bond breaking energy (E_b), and relaxation energy gain ($-E_r$) of the most stable SSV, SSSV, and SurDimer in the near-surface of reduced $CeO_2(111)$ with 5×5 periodicity.

Strain%	E_f			E_b			$-E_r$		
	SSV	SSSV	SurDimer/ Subdimer	SSV	SSSV	SurDimer/ SubDimer	SSV	SSSV	SurDimer/ Subdimer
+5	0.63	0.80	0.93	3.25	3.56	2.92	2.62	2.76	1.99
+4	0.91	1.02	1.16	3.42	3.73	3.04	2.51	2.71	1.88
+3	1.23	1.26	1.45	3.67	3.78	3.24	2.44	2.52	1.79
+2	1.49	1.47	1.69	3.85	3.89	3.31	2.36	2.42	1.62
+1	1.76	1.65	1.94	3.96	4.00	3.49	2.2	2.35	1.55
0	2.11	1.88	2.16/1.99	3.97	4.01	3.62/3.71	1.86	2.13	1.46/1.72
−1	2.38	2.11	2.44	4.08	4.11	3.72	1.70	2.00	1.28
−2	2.67	2.30	2.66	4.17	4.12	3.81	1.50	1.82	1.15
−3	2.93	2.49	2.85	4.38	4.25	3.91	1.45	1.76	1.06
−4	3.13	2.75	3.08	4.45	4.40	4.10	1.32	1.65	1.02
−5	3.45	2.89	3.23	4.49	4.35	4.19	1.04	1.46	0.96

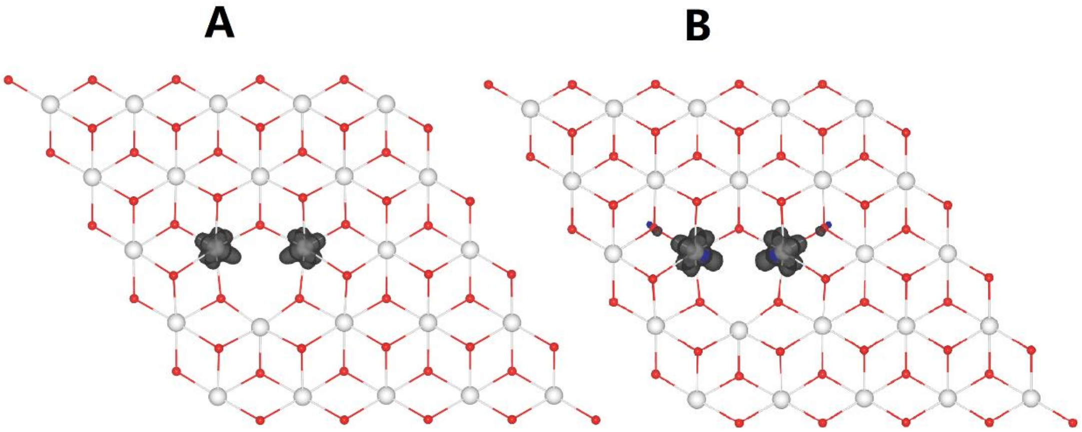

Figure S2. Isosurfaces of charge that fall into the (A) −0.5 eV to −0.28 eV energy range and (B) −0.28 eV to 0 eV for a SSV under −5% with AB Ce^{3+} configuration.

Table S10. O_{2p}-Ce_{5d}, the energy difference between the highest occupied 2*p*-states of O and the bottom of the empty 5*d*-states of Ce; O_{2p}-Ce_{4f}, the energy difference between the highest occupied 2*p*-states of O and the lowest occupied 4*f*-states of Ce; and *f*-splitting, the energy splitting between the two occupied 4*f* states of Ce for a SSV under –5%, 0% and +5% strain with different Ce^{3+} configurations, namely, AB, 12, and 1C with 5×5 periodicity.

SSV	O_{2p}-Ce_{5d}	O_{2p}-Ce_{4f}	*f* splitting
AB: –5%	4.85	0.78	0.00*
AB: 0%	4.97	0.81	0.00
AB: +5%	4.71	0.41	0.00
1C: –5%	4.91	0.74	0.06
1C: 0%	5.11	0.75	0.04
1C: +5%	4.68	0.30	0.16
12: –5%	4.99	0.89	0.00
12: 0%	5.11	0.80	0.00
12: +5%	4.73	0.31	0.00

* polarons are no longer fully localized in one specific Ce site, rather, the polaronic charge is shared between the two sites in a sort of bonding/anti-bonding configuration (cf. isosurfaces in Fig. S2).

Table S11. Oxygen vacancy formation energies (E_f), bond breaking energy (E_b), and relaxation energy gain ($-E_r$) of the most stable SSV, SSSV in the near-surface of reduced $CeO_2(111)$ with 2×2 periodicity.

Strain%	E_f		E_b		$-E_r$	
	SSV	SSSV	SSV	SSSV	SSV	SSSV
+5	1.06	0.99	3.13	3.27	2.07	2.28
+4	1.29	1.15	3.26	3.41	1.97	2.26
+3	1.53	1.32	3.36	3.54	1.83	2.22
+2	1.79	1.49	3.50	3.68	1.71	2.19
+1	2.04	1.67	3.65	3.78	1.61	2.11
0	2.30	1.84	3.76	3.86	1.46	2.02
−1	2.59	2.04	3.88	3.98	1.29	1.94
−2	2.87	2.22	4.00	4.05	1.13	1.83
−3	3.15	2.41	4.11	4.14	0.96	1.73
−4	3.42	2.57	4.22	4.17	0.80	1.60
−5	3.54	2.72	4.32	4.21	0.78	1.49

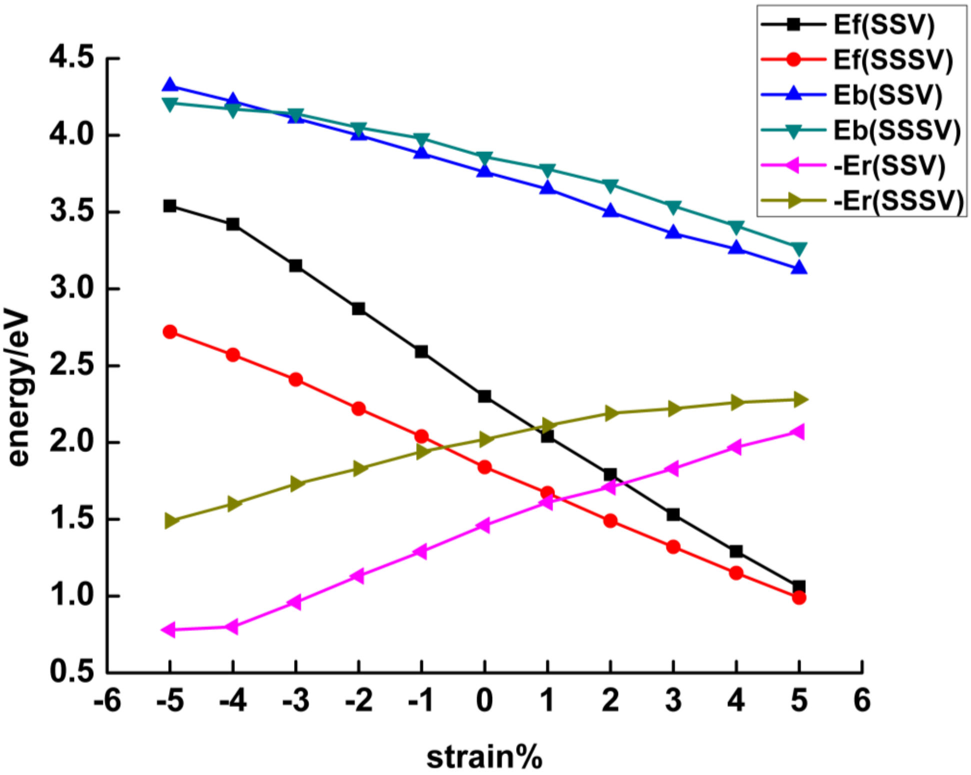

Figure S3. The near-surface oxygen vacancy formation energy, $E_f = E_b + E_r$, as a function of strain for the most stable SSV and SSSV with 2×2 periodicity. E_b is the energy cost to create a near-surface oxygen vacancy without allowing for lattice relaxations, i.e., the bond breaking energy, and E_r, the gain in relaxation energy.
